# ‘We may need some help; we are just parents who have chosen to engage in football’: a qualitative study on amateur coaches’ experiences of use of and support for injury prevention training in Sweden

**DOI:** 10.1136/ip-2024-045289

**Published:** 2024-07-18

**Authors:** Hanna Lindblom, Sofi Sonesson, Martin Hägglund

**Affiliations:** 1 Unit of Physiotherapy, Department of Health, Medicine and Caring Sciences, Linköping University, Linköping, Sweden; 2 Sport Without Injury ProgrammE (SWIPE), Department of Health, Medicine and Caring Sciences, Linköping University, Linköping, Sweden

**Keywords:** training, implementation / translation, qualitative research, sports / leisure facility

## Abstract

**Introduction:**

Achieving sufficient adherence with injury prevention exercise programmes is a challenge. The aim was to explore how amateur football coaches experience the use of and support for injury prevention training using the *Knee Control* programmes as examples.

**Methods:**

Semistructured interviews with 20 amateur football coaches around experiences of injury prevention training, facilitators and barriers, and perceived support, analysed with qualitative content analysis. Participants coached male and female, junior and senior teams. Experience of having used the *Knee Control* programmes was an inclusion criteria.

**Results:**

Four main categories were developed: *Why are we really doing this?*, *How do we make it work?*, *What are our drivers and challenges?* and *What could be improved?* Coaches were motivated for injury prevention training but faced challenges such as limited access to football grounds and low player motivation. To make the prevention programme work for them, they integrated it and used exercises in the pauses during football-specific drills, or used as a warm-up. Many conducted prevention routines from an early player age. Coaches believed preventive training use could be further enhanced by education and practical support, and by football associations and clubs working together to reduce injuries.

**Conclusion:**

Coaches were motivated and creatively worked with the prevention programme to make it fit their team. Even coaches with long-term experience of using prevention programmes wanted support, indicating that present implementation strategies targeting those about to start using prevention programmes should be complemented by continuous support for maintained use. These strategies should preferably target both coaches and players.

**WHAT IS ALREADY KNOWN ON THIS TOPIC**

**WHAT THIS STUDY ADDS**

- Coaches found ways to work around barriers to enable long-term use of IPEPs, such as integrating the programme into football training, scheduling prevention training in advance and sharing the responsibility of injury prevention between coaches in the team.
- Coaches were driven by different motivators, such as knowledge of the risks of injury and the positive effects of IPEPs.
- Coaches were challenged by factors such as their own competence and prerequisites, low player buy-in and external factors such as limited access to football grounds.

**HOW THIS STUDY MIGHT AFFECT RESEARCH, PRACTICE OR POLICY**

- Football associations and clubs working together with a vision to reduce injuries in football would signal to coaches and players that injury prevention is a priority.
- Use of IPEPs could be supported by knowledge and education in the programmes for coaches and players.
- Strategies need to be developed to support coaches who already use IPEPs, for example through giving examples on how to progress and vary training, and giving feedback on the player execution of the exercises and their leadership during preventive training.
- In addition to strategies aimed at coaches, players could be targeted with education and workshops to improve player understanding and buy-in.

## Introduction

 Injury prevention exercise programmes (IPEPs) efficaciously prevent injuries in football.^1^,^3^ However, coaches often modify the programme content or dosage, which may reduce preventive effectiveness.^4^,^6^ Reasons for these modifications include, for example, time constraints, limited space for the exercises, low player buy-in and poor programme fit.^5 6^ Successful implementation of IPEPs is a challenge, since it is dependent on behaviour change among coaches and athletes.^7^

We developed the Swedish IPEP *Knee Control* into *Knee Control+* as a response to requests for more variation and opportunities for progression from amateur coaches.^4 5^ *Knee Control* includes six main exercises, with four levels of progression and pair exercises. In total, the programme has 30 exercise options, targeting core stability, balance, jump-landing technique and proper knee alignment and was instructed to be used as warm-up. *Knee Control+* includes twice as many exercise options as *Knee Control*, and a more flexible set-up to better meet the coaches’ and players’ needs regardless of age or playing level.^8^ Similar initiatives have been seen for the IPEP *the 11*, that evolved into *the 11+*,^2^ to allow for inclusion of adductor strengthening exercises,^9^ the rescheduling of exercises within the programme^10^ and modifications to better align with the needs of goalkeepers^11^ and kids.^12^

The programmes are intended to be led by coaches. Behaviour change among coaches is necessary to start and to maintain use of IPEPs. The Health Action Process Approach (HAPA) model is a two-phase model that describes this behaviour change. In the first motivational phase, intention for change is formed based on the individual’s injury risk perceptions, outcome expectancies and action self-efficacy (belief in one’s ability to act). In the second goal-pursuit phase, this intention is translated into action supported by maintenance and recovery self-efficacy, action and coping planning^13 14^ and resulting in high or low adherence with an IPEP. Successful implementation of IPEPs may also be context-specific, dependent on factors such as the sport, the intended users and the level of play. Therefore, we need to know more about experiences of using IPEPs specifically in amateur football and focusing on the coaches, who, together with the players, are the intended users of IPEPs. Earlier studies have shown that the majority of amateur football coaches in Sweden are familiar with the *Knee Control* programmes and use them at least to some extent.^4 8^ A few qualitative studies have focused on coach experiences of injury prevention in sports, but targeted injury prevention in general^15^,^17^ or the *11+* specifically.^18^ Little is known about experiences of injury prevention training from the perspectives of amateur football coaches. Hence, the aim was to explore how amateur football coaches experience the use of and support for injury prevention training using the *Knee Control* programmes as examples.

## Methods

### Design

This was a qualitative study, with individual interviews of football coaches in one football district (Östergötland) in Sweden. We used a semistructured interview guide, which covered experiences of and support for injury prevention training. In this study, we had a constructionist perspective^19^ and aimed to capture and describe different perspectives and experiences of use of the *Knee Control* programmes and exemplify by using quotes from different coaches. The study was approved by the Swedish Ethical Review Authority (Dnr 2022-05430-01). The study has been checked against the consolidated criteria for reporting qualitative research checklist^20^ and all applicable criteria were met.

### Patient and public involvement

Coaches took part in the development of *Knee Control+*. We have had contact with coaches throughout the years during various studies, and in workshops and presentations, and thereby have had opportunities to learn from their experiences, which we used to shape the interview guide.

### Injury prevention context

The study took place in one football district where the *Knee Control* programmes have been actively disseminated since 2009 (*Knee Control*) and 2020 (*Knee Control+*), respectively, through distribution of programme material and workshops. During these years, the research group has conducted several studies on these IPEPs and has encountered many of the clubs in the district.^14 5 8 21^,^23^

### Study population and recruitment

Coaches for players ≥14 years of age and with experience of using the *Knee Control* or *Knee Control+* programmes were eligible. We used purposeful sampling and strived to select information-rich cases to illuminate the questions at hand.^19^ We strived for maximum variation of player age, sex (player and coach) and playing level, and teams situated in urban and rural areas. We intended to include 20 coaches, as this was perceived to be sufficient for the study’s aim. Contact information for coaches was collected from the clubs’ webpages, where player sex, age and level of play could also be retrieved. First, we sent an e-mail via a research group mail account with information about the study to strategically selected coaches (n=50). This was followed up by telephone contact with these coaches until 20 had agreed to take part and were scheduled for telephone interviews. In total, we had telephone contact with 30 coaches. Among those, 10 declined participation due to lack of interest, were no longer coaching or had no experience of the *Knee Control* programmes. Three of the 20 coaches had taken part in previous studies or workshops meaning they had had personal contact with the interviewer on a single occasion. Most coaches coached on a voluntary basis without compensation salary.

Eighteen of the coaches were male, two were female (mean age: 48.4 years, SD: 5.9). Nine coached male teams and 11 female teams; 10 were junior and 10 senior teams, 13 coached teams in urban areas and seven in rural areas. Coaches coached adolescent players or adult amateur male players in the third–ninth league (out of nine leagues) or amateur female players in the fourth–sixth league (out of six leagues). Nine of the coaches had experience of coaching both male and female teams. All had (at least) basic coach education. Six had taken part in education specifically about the *Knee Control* programmes. Coaches had a mean of 13 years of coach experience (range: 2–45 years).

### Data collection

Telephone interviews were performed by two female sports physiotherapists (first author, HL n=13, and second author, SS n=7), both PhD qualified, with previous experience in conducting qualitative studies and in using the *Knee Control* programmes. Interviews by telephone were chosen as this was convenient for the participants.^24^ Since the topic was not sensitive, we believed coaches would be able to fully express their views over telephone.^24 25^ Interviews took place from January to March 2023 and were recorded using a dictaphone. Coaches were free to decide on the time and location of the interviews. Verbal informed consent was collected from participating coaches in connection to the interviews. The interview guide was developed specifically for this study based on our experiences from previous studies on the *Knee Control* programmes^4 5 8 26^ and included questions on experiences of injury prevention training in general, and the *Knee Control* programmes in particular, facilitators and barriers, and perceived support for injury prevention training (online supplemental material). Questions were open-ended and worded to enable thoughtful responses about what was most important for the coaches.^19^ Additional probes were used when necessary to deepen the responses to a question.^19^ Before the interview, the interviewer presented herself by name, introduced herself as a researcher, and read the aim of the study aloud, but did not specifically describe her profession or expertise in the *Knee Control* programmes to enable the participants to speak freely about their views. Baseline demographics were also collected from the coaches before the qualitative interview with open-ended questions started. The interview guide was constructed by the authors (HL, SS, MH) and tested in two pilot interviews, one by each interviewer. After the pilot interviews, HL and SS discussed their experiences of the interviews and interview guide, and since both believed that the interview guide was sufficient for the study aim, and that their interview approach facilitated an open interview climate, no changes were made to the interview guide or interview approach. Both pilot interviews were included in the analysis. After the 20 interviews, the pseudonymised recordings were transcribed verbatim by a licensed transcription service. Transcripts or interpretations were not returned to the coaches to comment on.

### Analysis

We employed conventional qualitative content analysis of the transcribed interviews.^27^ Microsoft Excel was used to manage data. We followed the order below:

1. HL read and reread the interview transcripts to obtain an overall picture and made a short summary of each interview.
2. HL marked meaning units, significant parts of the text, that aligned with the study aim and used these for further analysis.
3. HL condensed meaning units and gave them short codes that illustrated their content.
4. HL sorted the codes into preliminary subcategories and categories that were internally homogenous and externally heterogenous, this entailed grouping codes with analogous content into the same category, while distinctly different content was allocated to separate categories.
5. HL made an analysis of the first interview, which was discussed with SS and MH. During the discussion, the researchers reflected on their interpretations to ascertain that these aligned with the interview transcripts.
6. Thereafter, HL analysed 10 interviews using the process described in points 1–4 above and made a preliminary categorisation that was discussed together with SS and MH.
7. HL then analysed the remaining interviews the same way and continuously refined and reorganised categories and subcategories to represent the data therein.
8. All authors (HL, SS, MH) met and agreed on the final categories and subcategories.
9. Finally, we made a negative case analysis where information within each category was critically revised to ensure that all codes had been correctly categorised and that there were no codes that contradicted or did not support the categories.

Since MH did not take part in the data collection but has extensive experience conducting research in football and experience as a football coach, he strengthened the researcher triangulation by being blank to the collected data. Additionally, by not taking part in data collection, he could make a negative case analysis without any preconceptions. After writing the results and discussion, we checked the main findings and interpretations against the short summaries of each interview, to ascertain that the results and interpretations were aligned with the immediate thoughts about each interview and that the results were based on the data and not on any author preconceptions.

Categories and subcategories are exemplified with quotations and presented together with the code (1–20) for each coach. Some quotations were shortened, which is indicated by […]. An example of a coding tree can be found in the online supplemental material.

## Results

**Figure 1:**
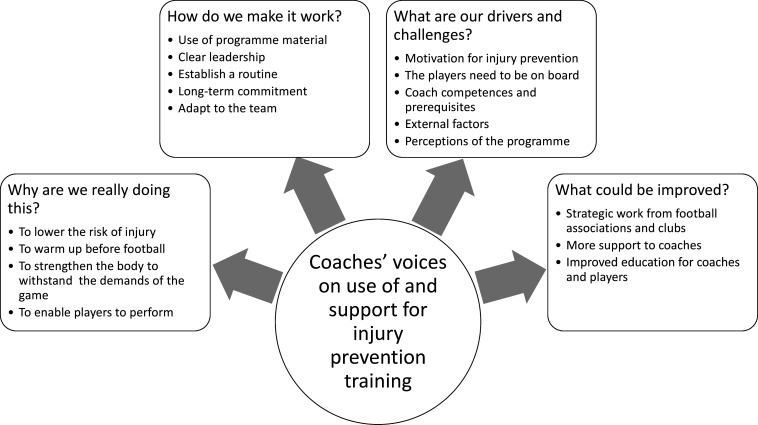
Illustration of the four categories and their subcategories.

The 20 interviews lasted between 27 and 60 min (mean 42 min). During the analysis, four main categories were developed (figure 1), which will be described in the following sections.

### Why are we really doing this?

Coaches described underlying reasons for using injury prevention training in four subcategories: ‘*To lower the risk of injury*’, ‘*To warm up before football*’, ‘*To strengthen the body to withstand the demands of the game*’ and ‘*To enable players to perform*’ (table 1). Coaches used injury prevention informed by their own injury or their team’s injury experiences. Coaches of female teams specifically mentioned a high prevalence of knee injuries in women as a reason for prevention. A few regarded injury prevention training mainly as a way of warming up. In addition to preventing injuries, injury prevention training was used to strengthen the body and offer versatile training as a complement to football training, or as preparation to strengthen the body before returning to play after injury or other absences. A holistic view of injury prevention was mentioned, covering sleep, nutrition, training load and recovery, to enable players to perform and benefit maximally from training.

**Table 1:** Illustration of quotes from the main category, *Why are we really doing this?*

ID	Meaning unit	Subcategory
4	Well, it is for a good reason that you do it. To try and minimise, and even eliminate, some injuries.	To lower the risk of injury
17	You can skip the warm-up because it is almost like a warm-up.	To warm up before football
3	You strengthen muscles in the whole body, both related to the knees and also the back.	To strengthen the body to withstand the demands of the game
10	Football is a very physical sport. You bump into each other, and I try to explain that you need to be as prepared as possible when you collide with another player.	
11	I think it is brilliant (injury prevention training) and an important part of enabling players to play the sport as much as they can.	To enable players to perform

ID refers to the coach ID within the study.

### How do we make it work?

The practical use of injury prevention was described in five subcategories: ‘*Use of programme material*’, ‘*Clear leadership*’, ‘*Establish a routine*’, ‘*Long-term commitment*’ and ‘*Adapt to the team*’ (table 2). Coaches used accessible printed and/or digital programme material as support when choosing exercises and as a trigger to use the programme, and drew inspiration from social media and various IPEPs. They shared responsibility for leading the preventive training with other coaches, divided responsibility between coaches or had a person with specific competencies who led the training. When implementing injury prevention training, they strived to make training a routine and planned preventive training in advance. They described different ways to make training fun and work for them, such as to integrate injury prevention training in the ordinary training, vary the exercises, and select a sample of exercises.

**Table 2:** Illustration of quotes from the main category, *How do we make it work?*

ID	Meaning unit	Subcategory
20	Even though I have used it a long time, I always take it out (the programme material). You always find something, that you have missed, that exercise. So, yes, I think it is excellent.	Use of programme material
2	The one who leads the training has planned today’s exercises. That person is well prepared and knows the exercises. Then he or she gathers the players in a ring when it is time for the exercises.	Clear leadership
14	Coaches need to help each other and lead the preventive training together, everyone who is around the team. It should be properly done before every training.	
1	If they start when they are seven, it will be a natural part of training. Then they are already stars at 13. That’s what you want to achieve, really.	Establish a routine
17	Early on, I decided that we should do this (injury prevention training). It is part of my plan. For me there is no competing agenda.	
9	We believe that if we do this long term and with perseverance, they won’t develop pain over time; everything is related.	Long-term commitment
1	You start with *Knee Control* with a few exercises. Then you practice with the ball, and then after 5 min you carry on with *Knee Control*. Then you repeat this two or three times. Somewhere around 5–15 min, depending on the weather.	Adapt to the team
12	We have included other exercises since we needed something new. The novelty, so to speak. We want to change a little.	

ID refers to the coach ID within the study.

### What are our drivers and challenges?

This category includes five subcategories: ‘*Motivation for injury prevention*’, ‘*The players need to be on board*’, ‘*Coach competences and prerequisites*’, ‘*External factors*’ and ‘*Perceptions of the programme*’ (table 3). Coaches were motivated by knowledge of the risk of injury and the positive effect of injury prevention training. Coaches perceived that they needed to make players understand the importance of injury prevention, but also believed players needed to take responsibility for their own health. Even though coaches had interest in and prioritised prevention, they were sometimes unsure about optimal training set-up and execution. Coaches are often the parents of players on the team, and serve in this role as volunteers, and were believed to face more challenges due to limited time and knowledge. External factors, predominantly access to football grounds on artificial turf during wintertime and the timing of training in relation to school or work schedules, could affect use of injury prevention. The *Knee Control* programmes were believed to be easy and versatile to use, and fit different target groups, but some eventually found them boring.

**Table 3:** Illustration of quotes from the main category, *What are our drivers and challenges?*

ID	Meaning unit	Subcategory
15	Among the girls that I have followed, only one in the whole team has actually suffered a serious knee injury. I hope and believe it is because we have been so meticulous about it (injury prevention training).	Motivation for injury prevention
5	It’s about making them realise that we do this for them. I can give them the tools, but they need to do the exercises properly to see results.	The players need to be on board
8	That feeling of uncertainty is often there. Is it enough, am I doing something unnecessary? Or am I even doing too much?	Coach competences and prerequisites
9	We may need some help; we are just parents who have chosen to engage in football.	
16	It is not a question of whether you want to or not. You have a job and family, and sometimes there is just not enough time when the demands on the training increase.	
12	You can’t just ‘waste’ half an hour and do it (injury prevention training) when you rent an expensive artificial turf ground. We do not have our own football ground on artificial turf. We always rent during this time of the year and we get very few hours.	External factors
15	It is really good that you get access to a compilation of exercises which facilitate variation. That makes it more fun. It may target exactly the same muscle group but we now do exercises differently compared to how we did them before.	Perceptions of the programme
19	It is really important that you push for it (injury prevention training) more. *Knee Control* is, not to sound negative, I have never felt that way, but I know coach colleagues who believe it is, more for girls. You may have forgotten that, wait a second, it is just as important for boys. I think this notion has been erased, at least in our club it has been.	

ID refers to the coach ID within the study.

### What could be improved?

Coaches’ suggestions on how to support both initiation of injury prevention training and long-term maintenance are described in three subcategories: ‘*Strategic work from football associations and clubs*’, ‘*More support for coaches*’ and ‘*Improved education for coaches and players*’ (table 4). Coaches wanted support from football associations and clubs to emphasise that injury prevention is a priority and to spread information about the programme. They also believed they would benefit from continuous reminders to do the preventive training, and evidence of the preventive effect. To have someone to discuss prevention with or help when starting up, progressing training or having someone who could lead training on a regular basis were also suggested. Coaches also believed that the first courses in the general coach education should include injury prevention training, and that players could be targeted with information and workshops about the programme.

**Table 4:** Illustration of quotes from the main category, *What could be improved?*

ID	Meaning unit	Subcategory
6	You need to start with it (injury prevention training) early (…). That the club somehow expresses that this is a target or something, to make all coaches work in the same direction, so to speak.	Strategic work from football associations and clubs
2	I can imagine that new coaches to boys’ and girls’ teams may need support and help to get started, and how to integrate it (injury prevention training) better into regular training.	More support to coaches
9	And we also need support sometimes. It is good when someone comes and takes a look at you and says, ‘now you’re sloppy with the knee angles’ or ‘now you’re doing four exercises that target the same muscle groups’.	
20	I really believe in specific contact persons whose task it is to follow-up the use of *Knee Control* and that it is being done, who perhaps could come to training and ensure that it is being used.	
7	(How would you like this education to be?) More practical. Physical meetings. That you get to test the exercises yourself and not only watch on a screen how they should be done.	Improved education for coaches and players
8	You could push for it (injury prevention training) more perhaps, during coach education and so. How important this is. (…) That you perhaps have a physiotherapist or someone who attends. Now, there are many men who are skilled at football, but they do not really know anything about injury prevention.	
20	I think that currently, with the Swedish Football Association, there are so many things that they communicate to us, but it does not really reach the players sometimes. When it comes to education and exercises that one can do, it is often only targeted towards us coaches.	

ID refers to the coach ID within the study.

## Discussion

The study showed that coaches were driven by different motivators, such as knowledge of the risks of injury and the effects of IPEPs, and that they regarded injury prevention training not only as a way to prevent injuries, but also to strengthen the body and enable players to perform well in football. Coaches were challenged by their own competence and prerequisites, low player buy-in and external factors, such as limited access to football grounds. Coaches had worked around barriers to enable long-term use of IPEPs, such as integrating the IPEP into football training, scheduling IPEP training in advance and sharing the responsibility of prevention with other coaches. However, more education and support for practical programme use was requested.

Most coaches used the *Knee Control* programmes to prevent injuries. To remain uninjured was seen as pertinent to being able to benefit optimally from preventive training, as well as being able to take part in football training and matches. Even though performance effects of the *Knee Control* programmes have not been shown,^21 22^ there may be an indirect effect on performance since injured players cannot benefit from football training and matches. A similar broad perspective on injury prevention, focusing on both training load and biopsychosocial aspects of health and injury, was described among Swedish high school coaches in different sports.^15^ In future efforts to promote injury prevention among less experienced coaches, we could present these different benefits of practicing preventive training to further motivate continuous IPEP use.

The coaches made training work in practice by sharing responsibility between coaches. They used easily accessible programme material as support and as a cue for programme use, and had a long-term perspective on, and planned for, progression and maintenance of the programme. They often integrated the programme in the football training, rather than using it in the more traditional way as a warm-up. This may be a time-efficient solution for teams with limited access to football grounds if using the exercises when getting ready for other exercises or instead of waiting for your turn during football-specific drills. Another strategy is to reschedule the preventive training, which has been suggested as improvement for the *11+*.^18^ Few coaches in the present study, however, reported rescheduling. Rescheduling of neuromuscular exercises to the end of training has been shown to improve player compliance and reduce injury burden.^10^ In *Knee Control+* programme material and during workshops, we suggested that the programme could be used before, during or after training, but we could extend this further by providing good examples on how to integrate the exercises and suggesting exercise variations that are particularly suitable when space is limited.

Overall, coaches were positive, and prioritised injury prevention training. They were motivated by the programme’s positive effects, indicating high intention according to the motivational phase of the HAPA model and fertile ground for IPEP use. In an earlier study, we identified suboptimal support in the HAPA goal-pursuit phase and a need to improve action and coping planning to facilitate long-term use.^28^ In the present study, coaches suggested action and coping planning strategies, that is, structuring plans for practically using the programme and coping with barriers for programme use, to achieve high adherence. First and foremost, coaches wanted to motivate the players and make them understand the benefits of injury prevention. Coaches suggested that programme material could be specifically targeted to players (and not only to coaches), and that workshops with experts in injury prevention training could be arranged for the whole team. Implementation and maintenance strategies could thus be better targeted to players in the future. We could also develop support specifically to aid the coaches with practical planning of how to use the IPEPs and structure coping plans based on known challenges with maintaining sufficient adherence over time. Coparticipation from coaches and players would be valuable in developing this implementation support. This would also be in line with research on other IPEPs and sports that have used project partnerships with both coaches and players, as well as other stakeholders, in codesigning IPEPs aiming to obtain high user buy-in and high adherence rates.^29^,^31^

Focusing on self-efficacy aspects, that are prominent in both phases of the HAPA model, collaboration with medical personnel came out as key to support use of injury prevention in a study on high school coaches,^15^ whereas coaches in the present study asked for support in general terms from ‘someone’ with more knowledge who could lead or plan preventive training, or plan for its progression. Coaches were uncertain whether they used the IPEP in the most optimal manner and referred to their limited knowledge. This was in line with a study in field hockey, where voluntary coaches held responsibility for injury prevention, even though they could not be expected to be experts, and the authors concluded that there is a need to support coaches more.^16^ We have also previously shown that coaches have low action self-efficacy, that is, low belief in their ability to use IPEPs, and improved self-efficacy would be valuable.^5^ One way to support coaches could be to offer education to health professionals and other relevant stakeholders, such as fitness coaches, to increase their expertise in injury prevention and make them able to ‘train the trainers’. For most clubs at amateur level, it is unfeasible to hire someone to lead the training on a regular basis. However, arranging on demand consultations with an injury prevention expert, for example, a specially trained fitness coach or physiotherapist, could be a step forward.

### Strengths and limitations

When interpreting the findings, study trustworthiness must be considered. We refer to the four criteria of trustworthiness originally presented by Guba and Lincoln^32^: credibility, transferability, dependability and confirmability, and their application as described by Shenton.^33^ To strengthen study credibility, we conducted a negative case analysis and specifically searched for data that did not fit into the present categories. This was done between all three researchers, adding the opportunity for peer scrutiny and triangulation.^33^ We also compared summaries of the meaning in each interview with the end result, and made slight adjustments to ensure that the findings were consistent with the overall feeling during interviews. Even though only one author coded the data, all authors had access to a complete coding tree with all original codes and valued and interpreted these until all agreed on the final categorisation. A limitation with the study was that almost all included coaches had long-term experience of injury prevention programme use, whereas the perspective of a new user who is about to, or has just adopted an injury prevention programme is under-represented. Coaches who had not adopted an IPEP would obviously not have experience of its use and could not take part. Since most coaches were long-time users, this implies that they had found solutions to work around barriers for programme use. Hence, findings are mainly valuable for the concrete examples of solutions to facilitate continuous programme use.

Another limitation inherent in the qualitative design is the question of transferability. Results might be transferred to similar contexts, predominantly amateur football with coaches working on a voluntary basis, and regarding use of similar IPEPs. Few participants were female, which corresponds with the sex distribution in football teams, and was therefore not believed to affect transferability. The 20 interviews with coaches representing different perspectives were believed to be sufficient to respond to the study’s aim considering the richness and quality of data and the phenomenon under study.^34^ For assessment of dependability and confirmability, we strived for in-depth methodological descriptions and peer-triangulation between researchers to ascertain neutrality to the findings. The illustration of the categorisation, from meaning unit to subcategories and main categories, also enables the reader to determine confirmability.

## Conclusion

Coaches were motivated and overcame challenges using strategies such as integrating the IPEP into football training, scheduling preventive training in advance and sharing the responsibility of prevention. Even coaches with long-term experience of using IPEPs expressed a need for support. This indicated that present implementation strategies with workshops and programme material targeting those about to start using IPEPs should be complemented with strategies to support maintained IPEP use, as well as strategies that target players specifically.

## Supplementary material

10.1136/ip-2024-045289 online supplemental file 1

## Data Availability

All data relevant to the study are included in the article or uploaded as supplementary information.

## References

[1] Waldén M, Atroshi I, Magnusson H (2012). Prevention of acute knee injuries in adolescent female football players: cluster randomised controlled trial. BMJ.

[2] Soligard T, Myklebust G, Steffen K (2008). Comprehensive warm-up programme to prevent injuries in young female footballers: cluster randomised controlled trial. BMJ.

[3] Emery CA, Meeuwisse WH (2010). The effectiveness of a neuromuscular prevention strategy to reduce injuries in youth soccer: a cluster-randomised controlled trial. Br J Sports Med.

[4] Lindblom H, Waldén M, Carlfjord S (2014). Implementation of a neuromuscular training programme in female adolescent football: 3-year follow-up study after a randomised controlled trial. Br J Sports Med.

[5] Lindblom H, Carlfjord S, Hägglund M (2018). Adoption and use of an injury prevention exercise program in female football: a qualitative study among coaches. Scand J Med Sci Sports.

[6] Owoeye OBA, Emery CA, Befus K (2020). How much, how often, how well? Adherence to a neuromuscular training warm-up injury prevention program in youth basketball. J Sports Sci.

[7] Verhagen E, Bolling C (2018). We dare to ask new questions. Are we also brave enough to change our approaches?. Transl Sports Med.

[8] Lindblom H, Sonesson S, Forslind J (2023). Implementation of the injury prevention exercise programme *Knee Control+*: a cross-sectional study after dissemination efforts within a football district. Inj Prev.

[9] Harøy J, Thorborg K, Serner A (2017). Including the Copenhagen adduction exercise in the FIFA 11+ provides missing eccentric hip adduction strength effect in male soccer players: a randomized controlled trial. Am J Sports Med.

[10] Whalan M, Lovell R, Steele JR (2019). Rescheduling part 2 of the 11+ reduces injury burden and increases compliance in semi-professional football. Scand J Med Sci Sports.

[11] Al Attar WSA, Faude O, Bizzini M (2021). The FIFA 11+ shoulder injury prevention program was effective in reducing upper extremity injuries among soccer goalkeepers: a randomized controlled trial. Am J Sports Med.

[12] Rössler R, Junge A, Bizzini M (2018). A multinational cluster randomised controlled trial to assess the efficacy of ‘11+ Kids’: a warm-up programme to prevent injuries in children’s football. Sports Med.

[13] Schwarzer R (2016). Health Action Process Approach (HAPA) as a theoretical framework to understand behavior change. AP.

[14] Zhang C-Q, Zhang R, Schwarzer R (2019). A meta-analysis of the health action process approach. Health Psychol.

[15] Kempe H, Rasmussen-Barr E, von Rosen P (2023). Coaches’ experiences of injury prevention in youth elite athletes: an interview study of 10 coaches. Phys Ther Sport.

[16] Rees H, Matthews J, McCarthy Persson U (2021). Coaches’ attitudes to injury and injury prevention: a qualitative study of Irish field hockey coaches. BMJ Open Sport Exerc Med.

[17] Møller M, Zebis MK, Myklebust G (2021). “Is it fun and does it enhance my performance?” - key implementation considerations for injury prevention programs in youth handball. J Sci Med Sport.

[18] Winstanley C, Reid D, Fulcher ML (2023). Suggested improvements to the 11+ as identified by coaches, players, strength and conditioning staff and medical staff in New Zealand football. BMJ Open Sport Exerc Med.

[19] Patton MQ (2015). Qualitative research & evaluation methods.

[20] Tong A, Sainsbury P, Craig J (2007). Consolidated criteria for reporting qualitative research (COREQ): a 32-item checklist for interviews and focus groups. Int J Qual Health Care.

[21] Lindblom H, Waldén M, Hägglund M (2012). No effect on performance tests from a neuromuscular warm-up programme in youth female football: a randomised controlled trial. Knee Surg Sports Traumatol Arthrosc.

[22] Lindblom H, Waldén M, Hägglund M (2020). Performance effects with injury prevention exercise programmes in male youth football players: a randomised trial comparing two interventions. Sports Med Open.

[23] Lindblom H, Sonesson S, Torvaldsson K (2023). Extended *Knee Control* programme lowers weekly hamstring, knee and ankle injury prevalence compared with an adductor strength programme or self-selected injury prevention exercises in adolescent and adult amateur football players: a two-armed cluster-randomised trial with an additional comparison arm. Br J Sports Med.

[24] Ward K, Gott M, Hoare K (2015). Participants’ views of telephone interviews within a grounded theory study. J Adv Nurs.

[25] Sturges JE, Hanrahan KJ (2004). Comparing telephone and face-to-face qualitative interviewing: a research note. Qual Res.

[26] Lindblom H, Waldén M, Hägglund M (2023). Adherence to injury prevention exercise programmes in amateur adolescent and adult football: a detailed description of programme use from a randomised study. Sports Med Open.

[27] Hsieh HF, Shannon SE (2005). Three approaches to qualitative content analysis. Qual Health Res.

[28] Lindblom H, Hägglund M (2024). Motivation and goal-pursuit for injury prevention training in amateur football coaches: a cross-sectional study using the Health Action Process Approach. Inj Prev.

[29] Bruder AM, Donaldson A, Mosler AB (2023). Creating Prep to Play PRO for women playing elite Australian football: a how-to-guide for developing injury-prevention programs. J Sport Health Sci.

[30] Ageberg E, Bunke S, Linnéll J (2024). Co-creating holistic injury prevention training for youth handball: development of an intervention targeting end-users at the individual, team, and organizational levels. BMC Sports Sci Med Rehabil.

[31] Bruder AM, Patterson BE, Crossley KM (2024). If we build it together, will they use it? A mixed-methods study evaluating the implementation of Prep-to-Play PRO: an injury prevention programme for women’s elite Australian football. Br J Sports Med.

[32] Guba E, Lincoln Y (1981). Effective evaluation: improving the usefulness of evaluation results through responsive and naturalistic approaches.

[33] Shenton AK (2004). Strategies for ensuring trustworthiness in qualitative research projects. EFI.

[34] Vasileiou K, Barnett J, Thorpe S (2018). Characterising and justifying sample size sufficiency in interview-based studies: systematic analysis of qualitative health research over a 15-year period. BMC Med Res Methodol.

