# Supplementary material for: ‘We may need some help; we are just parents who have chosen to engage in football’: a qualitative study on amateur coaches’ experiences of use of and support for injury prevention training in Sweden
Source: Inj Prev. 2024 Jul 18;31(6):e045289. doi: 10.1136/ip-2024-045289 (PMC12703322; doi:10.1136/ip-2024-045289)
Supplement: online supplemental file 1 [file ip-31-6-s001.pdf]

Supplementary material

Interview guide

Key questions on injury risk and implementation of injury prevention programmes

1. Please describe your experiences of football related injuries to players in your team or other teams.

2. What do you think about injury prevention training?
    a. Please describe your experiences of injury prevention training
    b. What pros/cons do you see with injury prevention training programmes?
    c. Please describe your experiences of pain in adjunction with injury prevention training.

Now let's move on to discuss *Knee Control* and *Knee Control+*, specifically.

3. What is your opinion about *Knee Control/Knee Control+*?
    a) What is your opinion about the programme material, for example the printed or digital folder, and how to access and use the programme?
    b) What is your opinion about the exercises in *Knee Control/Knee Control+*?
    c) What do you think about the programme set-up in *Knee Control/Knee Control+*?

4. What might facilitate use of *Knee Control/Knee Control+*, or make it more difficult?
    a) What support do you require when using *Knee Control/Knee Control+* in your team?
    b) What are your thoughts on education about *Knee Control/Knee Control+* for you as a coach or for players?
    c) Are there any other ways to support the use of *Knee Control/Knee Control+*, in your opinion?

5. Do you have any additional thoughts about injuries or injury prevention measures that you would like to share?

Probes

• Please explain more

• Can you give an example…?

Main questions (1-5) were asked to all coaches, sub-questions (a-c) and probes were asked when necessary to deepen the answers.

Example of a coding tree

Meaning unit	Code	Sub-category	Category
You strengthen muscles in the whole body, both related to the knees and also the back.	All-round training	To strengthen the body to withstand the demands of the game	Why are we really doing this?
Football is a very physical sport. You bump into each other, and I try to explain that you need to be as prepared as possible when you collide with another player.	Endure load	To strengthen the body to withstand the demands of the game	Why are we really doing this?
I can imagine that new coaches to boys' and girls' teams may need support and help to get started, and how to integrate it better into regular training.	Support during the start	More support to coaches	What could be improved?
And we also need support sometimes. It is good when someone comes and takes a look at you and says, "now you're sloppy with the knee angles" or "now you're doing four exercises that target the same muscle groups".	Someone who guides you	More support to coaches	What could be improved?
I really believe in specific contact persons whose task it is to follow up the use of *Knee Control* and that it is being done. Who perhaps could come to training and ensure that it is being used.	Control and reminders	More support to coaches	What could be improved?
